# Engineered *In Vitro* Models of Tumor Dormancy and Reactivation

**DOI:** 10.1186/s13036-018-0120-9

**Published:** 2018-12-27

**Authors:** Shantanu Pradhan, John L. Sperduto, Cindy J. Farino, John H. Slater

**Affiliations:** 1 Department of Biomedical Engineering, University of Delaware, 150 Academy Street, 161 Colburn Lab, Newark, DE 19716 USA; 2 Delaware Biotechnology Institute, 15 Innovation Way, Newark, DE 19711 USA; 3 Department of Materials Science and Engineering, University of Delaware, 201 DuPont Hall, Newark, DE 19716 USA

**Keywords:** Extracellular matrix, Single cell dormancy, Angiogenic dormancy, Quiescence, Metastasis, Relapse, Hypoxia, Drug Testing, Tissue Engineering, Microphysiological Systems

## Abstract

Metastatic recurrence is a major hurdle to overcome for successful control of cancer-associated death. Residual tumor cells in the primary site, or disseminated tumor cells in secondary sites, can lie in a dormant state for long time periods, years to decades, before being reactivated into a proliferative growth state. The microenvironmental signals and biological mechanisms that mediate the fate of disseminated cancer cells with respect to cell death, single cell dormancy, tumor mass dormancy and metastatic growth, as well as the factors that induce reactivation, are discussed in this review. Emphasis is placed on engineered, *in vitro*, biomaterial-based approaches to model tumor dormancy and subsequent reactivation, with a focus on the roles of extracellular matrix, secondary cell types, biochemical signaling and drug treatment. A brief perspective of molecular targets and treatment approaches for dormant tumors is also presented. Advances in tissue-engineered platforms to induce, model, and monitor tumor dormancy and reactivation may provide much needed insight into the regulation of these processes and serve as drug discovery and testing platforms.

## Introduction

Metastasis is responsible for the vast majority of cancer-related deaths worldwide, with one study estimating a dismal 5-year survival rate of only 26% for metastatic breast cancer patients [1–5]. Even though advances in early detection, diagnosis, and treatment of cancer have significantly improved patient outcome and survival, treatment of metastatic disease is still challenging, with only palliative options available in many cases. A major roadblock in the prevention and treatment of metastasis stems from a lack of understanding of the molecular mechanisms driving metastatic recurrence. This in part stems from the high degree of inter-tumoral and intra-tumoral heterogeneity, making it difficult to predict treatment outcomes. Cancer recurrence post-surgery, and after termination of therapy, has been a commonly observed problem across many cancer types [1, 6–10]. Patients diagnosed at an early stage with small tumors, and no lymphatic presence, have a 25-30% chance of recurrence after 10-15 years [11]. Analysis of long-term survival outcomes of patients suggests that the probability of metastatic recurrence and death follows two distinct peaks: one at 1-2 years and another at 5 years post-surgery. Early detection and adjuvant chemotherapy provide some prevention for early relapse but neither approach is effective at preventing relapse after 5 years [11–13].

Over the past few decades, researchers have postulated and demonstrated the presence of residual and disseminated tumor cells in patients that undergo a period of latency or dormancy [6, 14–17]. This latency period can range from a few months to as long as decades, depending on the cancer subtype, molecular characteristics and receptor status, patient lifestyle, systemic inflammation and a host of other factors [9, 16–20]. However, upon being stimulated by specific microenvironmental factors, these dormant cells can become activated, form micrometastases, and eventually macrometastases, often with increased chemoresistance, leading to poor patient outcome and reduced survival [20–22]. Hence, preemptively targeting dormant tumor cells offers a potential window of opportunity for prevention of metastatic relapse in patients.

This review provides an overview of engineered, *in vitro*, models that have been developed to investigate the roles that microenvironmental factors play in inducing and regulating tumor dormancy. Microenvironmental factors that induce, regulate, and maintain tumor dormancy are classified into four subgroups: 1) extracellular matrix (ECM), 2) signaling from secondary cell types, 3) biochemical factors and 4) drug treatment, and their distinct roles are summarily described. Engineered models developed to investigate escape from dormancy through reactivation and for identifying and testing potential drug candidates are also reviewed. It is hoped that the clinical challenges related to tumor dormancy gain wider attention in the biomaterials and tissue engineering communities, to focus efforts toward development of advanced recapitulative models of the dormant tumor niche, and for identifying dormancy-associated targets for drug development.

## Tumor dormancy

The temporal progression of metastasis starting with cell escape from the primary tumor and resulting in secondary tumors in foreign tissue is termed the ‘metastatic cascade’. Cells originating from a primary tumor can invade the surrounding tissue, intravasate into nearby blood vessels, travel through systemic vasculature as circulating tumor cells (CTCs), extravasate into secondary tissues (e.g. brain, liver, lung, bone marrow), and form metastases [23, 24]. The hematogenous metastatic process is extremely inefficient as only a small percentage of disseminated tumor cells form metastases [25–28]. Clinical studies of metastatic recurrence and mathematical modeling of tumor regrowth kinetics indicate that disseminated tumor cells may lie dormant for extended periods of time prior to being stimulated into an active growth state [17, 19, 29–33]. Additionally, tumor cells may disseminate early from a primary tumor (which is still clinically undetectable) and appear as metastatic tumors in secondary organs prior to detection of the primary tumor, leading to classification as tumors of unknown origin [34, 35]. These occult indolent tumors may lie dormant throughout the lifetime of the patient, primarily due to immune regulation [21, 36–38]. Interestingly, the primary tumor is also hypothesized to create ‘stress microenvironments’ for disseminated tumor cells by stimulating systemic immunoregulatory action and subsequently preventing dormant tumor cells from being activated [35, 39–41].

**Fig. 1:**
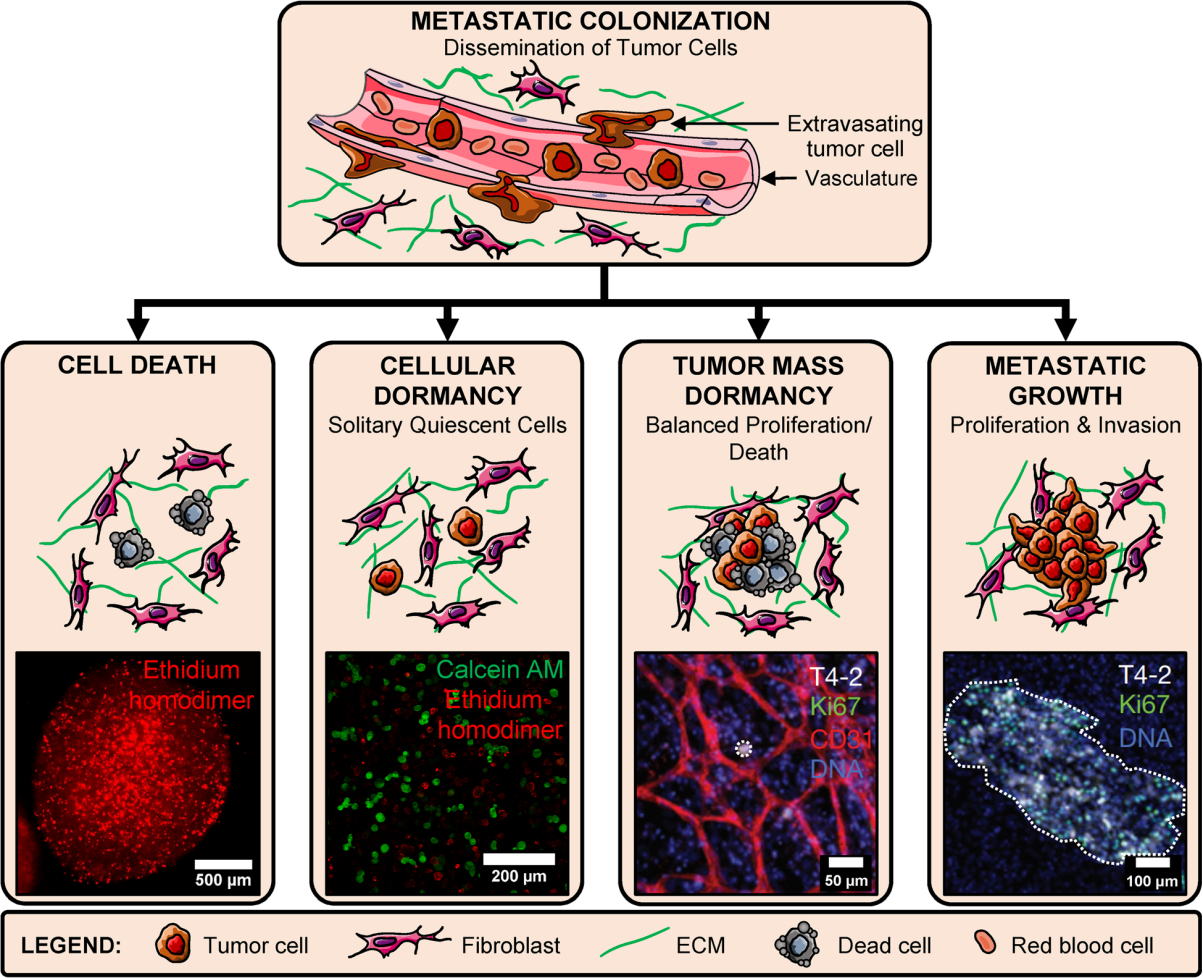
Fate of disseminated tumor cells. Circulating tumor cells extravasate from vasculature at secondary sites and undergo one of four fates in the secondary niche: cell death (primarily via apoptosis), cellular dormancy (remain as single quiescent cells), tumor mass dormancy (small clusters with balanced proliferation and apoptosis) and metastatic growth (high proliferation and invasion). Cell Death: representative image of MCF7 cancer cells within hydrogel millibeads fluorescently labeled with ethidium homodimer (red) (Adapted from [90]) Copyright 2014, ACS. Cellular Dormancy: representative image of MDA-MB-231 breast cancer cells within hydrogels fluorescently labeled with calcein AM (green)/ethidium homodimer (red) (unpublished). Tumor Mass Dormancy: HMT-3522-T4-2 breast cancer cells cultured with lung stromal cells and endothelial cells form a small, non-proliferative colony (dotted circle) (Adapted from [42]). Metastatic Growth: HMT-3522-T4-2 cells cultured with lung stromal cells develop into invasive, proliferative clusters representative of metastatic outgrowth (dotted region) (Adapted from [42]). Copyright 2013, Springer Nature

Various scenarios concerning the fate of extravasated tumor cells have been proposed and validated using *in vivo* models [16, 42]. These scenarios describe the existence and persistence of dormant tumor cells in secondary niches along with a multitude of factors (signaling from secondary cell types, ECM properties, and biochemical factors), some of which induce cell quiescence and cancer latency. Multiple theories concerning the prevalence of one scenario over others have been proposed, but in reality, the co-existence of these scenarios in parallel is quite likely; although not yet definitively demonstrated in clinical studies [30, 43]. These scenarios are presented as potential fates which disseminated cells may undergo in secondary niches either through tumor-intrinsic or tumor-extrinsic pathways (Fig. 1).

### Cell death

A majority of disseminated cells die either in the systemic cardiovasculature or after extravasation into secondary tissue. Death of CTCs during circulation is chiefly mediated by vascular stress and immunomodulatory mechanisms of macrophages, leukocytes, and platelets, resulting in a short half-life of only 2-3 hours [17, 19, 44]. CTCs that do survive, and are able to colonize secondary tissue, face additional microenvironmental stress and immunomodulatory suppression in the complex milieu, which is generally very different from the primary tumor niche [17, 25, 45]. Hence, death via apoptosis and anoikis is common in a majority of disseminated cells [25, 46]. Interestingly, some ovarian cancer cells have been observed to use autophagy-related mechanisms to survive as dormant cells in the *in vivo* tumor microenvironment [47].

### Cellular dormancy

A majority of surviving cells in the dormant niche are believed to survive as single cells with G_0_ cell cycle arrest, altered metabolic profiles and induction of anti-apoptotic cell survival mechanisms [25, 48–50]. The presence of persistent single tumor cells in various secondary niches (e.g. bone marrow, brain perivascular niche) has been experimentally observed in *in vivo* models and in human subjects with no clinically detectable disease [19, 51, 52]. The intrinsic and extrinsic factors that support this population of dormant cells for extended time periods have only been recently explored, although much progress is needed in determining and identifying the potential of these single cells toward activation and tumor growth [11, 21, 34, 53–55]. Evolutionary theories posit that complete eradication of these dormant cells may be too far-fetched; however, efforts to induce and maintain the cells in a dormant state for long time periods are currently being explored [34].

### Tumor mass dormancy

In addition to dormant single cells, small cell clusters maintaining a delicate balance between proliferation and apoptosis may occur in a manner that prevents tumor growth. These small clusters are often discounted as dysplastic local tissue [56]. Small cell clusters in balanced dormancy contain low proliferation and a mix of pro-angiogenic and anti-angiogenic stromal and cellular cues that balance each other to maintain tumoral homeostasis [11, 34, 36]. This state is also referred to as balanced population dormancy and can be further sub-divided into: 1) immune-suppressed dormancy (mediated by persistent cytotoxic activity of immune cells to restrict tumor growth) and 2) pre-angiogenic dormancy (caused by a lack of angiogenic signaling and deficiency of nutrients, characterized by avascular and whitish masses) [11, 49, 50, 57, 58]. In some cases, these clusters may become larger than 1-2 mm without vascularization and form distinct central necrotic cores. These small tumor masses have been demonstrated to harbor a pool of stem cells which undergo asymmetric cell division to maintain a balance of proliferative and apoptotic cells [59, 60]. A number of studies demonstrating the presence and temporal evolution of avascular dormant tumors have been conducted to investigate the role of microenvironmental factors regulating this dormancy state [61–64]. However, competing theories suggest that population dormancy is much rarer than single cell dormancy and may possibly be a temporal step of single dormant tumor cells heading toward metastatic outgrowth [29, 65].

### Metastatic outgrowth

Dormant single cells or avascular cell clusters can be triggered toward aggressive and invasive growth upon stimulation by various factors including angiogenic sprouting, inflammatory cytokines, aberrations in stromal cues, and others [21, 22, 42, 66]. This change in state often leads to metastatic colonization, inhibition of secondary organ function, and is the prime cause of metastatic relapse and death among patients. Metastatic relapse has been studied extensively in animal models and current efforts are directed toward prevention or delay of this phenomenon to increase patient survivability [9, 62, 63, 67].

The molecular and genetic mechanisms underlying cellular dormancy, tumor mass dormancy, and tumor cell survival in dormancy-inducing niches, as well as reactivation, have been extensively reviewed previously [18, 25, 26, 45, 49, 68–76]. Integrin engagement of dormant tumor cells with the surrounding ECM has been implicated in maintaining cellular quiescence [20, 77, 78]. Biological observations of tumor dormancy have mostly been restricted to animal models, due to a lack of well-defined *in vitro* models [54, 57, 79, 80]. While animal models provide a high degree of physiological context, they entail several limitations with respect to investigating dormancy [11, 43, 81]. Longitudinal detection, observation and fate-tracking of single tumor cells or small cell clusters simultaneously within multiple organs of a complex organism is severely restricted by current imaging limitations, although some advances are being made in this aspect [82–84]. The choice of cell lines for investigating dormancy *in vivo* is not appropriately classified yet; aggressive cell lines in two-dimensional (2D) culture may form overt macrometastases in animals within a shorter time frame than what may be required to study long-term dormancy, while cell lines ideal for studying dormancy may be misclassified as non-malignant or non-tumorigenic [11]. Additionally, inducing spontaneous dormancy in animals is difficult due to the stochastic nature of metastasis and tumor growth [81]. Most of the knowledge concerning *in vivo* dormancy has been obtained from histological analysis, using chick chorioallantoic membrane (CAM) models or models using superficial anatomic sites where the cell fate can be tracked which is often difficult for internal organs [11, 36, 64, 85]. Engineered, *in vitro* models may provide a means to overcome some of the limitations associated with animal studies while also providing more control over the parameters thought, or known, to induce dormancy. Recent efforts to implement engineered models to induce, model, and investigate the roles of microenvironmental factors in these processes are discussed in the following sections.

## *In vitro* approaches to model tumor dormancy

Current efforts in tissue engineering to generate cancer models are often implemented to investigate the metastatic cascade, recapitulate the aberrant tumor microenvironment, for biophysical and biochemical regulation of cancer cell behavior, and for drug development. However, *in vitro* models to investigate dormancy are far fewer in number [86]. One of the reasons for the paucity in dormancy models is the lack of a definitive roadmap for analysis, classification and characterization of dormant cell behavior spanning multiple cancer types, as well as establishment of well-defined dormancy metrics. However, with advances in dormancy biology and in biomaterial, biofabrication and microfluidic technologies, novel *in vitro* dormancy models are being developed (Table 1). These models are expected to provide deeper insight into the molecular mechanisms regulating dormancy while providing facile, higher-throughput and well-controlled microenvironments for drug discovery.

**Table 1:** Summary of *in vitro* dormancy models classified by cancer/cell type and mode of dormancy induction with associated metrics used to determine dormancy status

Cancer/Cell Type	Mode of Dormancy Induction	Metrics Analyzed
Breast Cancer - MDA-MB-231 [43, 59, 82, 88, 91, 93, 96, 98, 106, 116, 118–120, 127, 128, 132, 135–137, 156, 179] - MDA-MB-231BRMS1 [139] - MCF-7 [43, 44, 59, 82, 89, 93, 98, 118, 128, 132, 134–137, 139, 176] - T47D [95, 121, 133, 135, 138, 177, 179, 181] - MDA-MB-435 [58, 176] - MDA-MB-468 [133] - MDA-MB-453 [58] - ZR-75-1 [58] - SUM149 [58] - SUM159 [58, 148] - BT474 [58] - D2.0R [162, 163, 196] - 4T1 [176]	ECM-Induced - Matrix Stiffness/Physical Confinement [87–90, 92] - Matrix Composition/ Architecture [92, 97, 105] - Integrin Engagement [135, 137, 138, 162, 163, 196] Cell Signaling-Induced - Endothelial Cells [42, 58, 81, 105, 119] - Hepatocytes/NPCs [81, 105, 119] - MSCs [42, 58, 117, 122, 179] - Fibroblasts [42, 58, 120] - Osteoblasts [58, 139] - Exosomes/EVs [121, 122] Biochemical-Induced - Hypoxia [128, 129, 133] - FGF-2 [135–138, 140, 181] - Thrombospondin [42] Drug-Induced - Doxorubicin [81, 148] - Docetaxel [148] - Carboplatin [95]	- Proliferation [42, 58, 81, 87, 88, 95, 105, 117, 119, 129, 148, 163, 172] - Cell Cycle Analysis [95, 117, 120, 121, 179] - Metabolic Activity [88, 89, 95, 119, 133] - Viability [87–90, 95, 120, 181] - Morphology [43, 91, 93, 98, 119, 127, 138, 139, 162, 177, 196] - Gene/Protein Expression [42, 81, 95, 105, 117, 119, 120, 133, 135–138, 140, 162, 172, 196] - Invasion/Motility [42, 87, 95, 97] - Chemoresistance [89, 128, 172, 177] - Stem Cell Expression [95, 117, 122]
Prostate Cancer - PC-3 [117, 118, 120, 176] - DU145 [148, 176, 177] - C4-2B [128] - LnCAP [88]	ECM-Induced - Matrix Stiffness/Physical Confinement [88] Cell Signaling-Induced - Prostate Stromal Cells [120] - Endothelial Cells [118] - MSCs [117, 118] Biochemical-Induced - Hypoxia [128] Drug-Induced - Docetaxel [148]	- Proliferation [118, 148] - Metabolic Activity [88] - Morphology [117, 118, 128] - Chemoresistance [176, 177]
Lung cancer - A549 [94, 117, 178] - PC-9 [157] - H1975 [104]	ECM-Induced - Physical confinement [94] - Mechanical forces [104] Cell Signaling-Induced - MSCs [117]	- Proliferation [104] - Cell Cycle Analysis [94] - Morphology [94, 104, 117] - Gene/Protein Expression [94, 104, 157, 178] - Invasion/Motility [94, 104] - Chemoresistance [94, 104, 157, 178]
Colorectal/Colon Cancer - DLD-1 [177] - LoVo [147] - HCT-116 [89, 128, 147] - Primary colon cancer cells [147, 177]	ECM-Induced - Matrix Stiffness/Physical Confinement [89] Biochemical-Induced - Hypoxia [128] - EGF [147] Drug-Induced - 5-FU [147]	- Proliferation [147] - Metabolic Activity [89, 128] - Viability [89, 177] - Morphology [89, 128] - Chemoresistance [89, 128, 177]
Pancreatic Cancer - PANC-1 [117] - Capan-1 [176] - CFPAC [89]	ECM-Induced - Matrix Stiffness/Physical Confinement [89] Cell Signaling-Induced - MSCs [117]	- Proliferation [89] - Metabolic Activity [89] - Viability [89] - Morphology [89, 117] - Chemoresistance [89, 176]
Other Cancers - Bladder Cancer (T24, UMUC-3, J82) [120, 176] - Oral Squamous Cell Carcinoma (SCC-71) [128] - Osteosarcoma (U2OS, MG63, Saos-2) [58, 128] - Gastric Cancer (AGS) [176] - Glioblastoma (U251) [176] - Ovarian Cancer (OVCAR-5) [88]	ECM-Induced - Matrix Stiffness/Physical Confinement [88, 128] Cell Signaling-Induced - Endothelial Cells [58] - MSCs [58] - Osteoblasts [58] - Prostate Stromal Cells [120] - Fibroblasts [120]	- Proliferation [58, 128] - Cell Cycle Analysis [120] - Metabolic Activity [88, 128] - Viability [88, 120] - Chemoresistance [128, 176]

Abbreviations: *5-FU* 5-Fluorouracil, *ECM* Extra-Cellular Matrix, *EGF* Epidermal Growth Factor, *EVs* Extracellular Vesicles, *FGF-2* Fibroblast Growth Factor-2, *MSCs* Mesenchymal Stem/Stromal Cells, *NPCs* Non-Parenchymal Cells

**Fig. 2:**
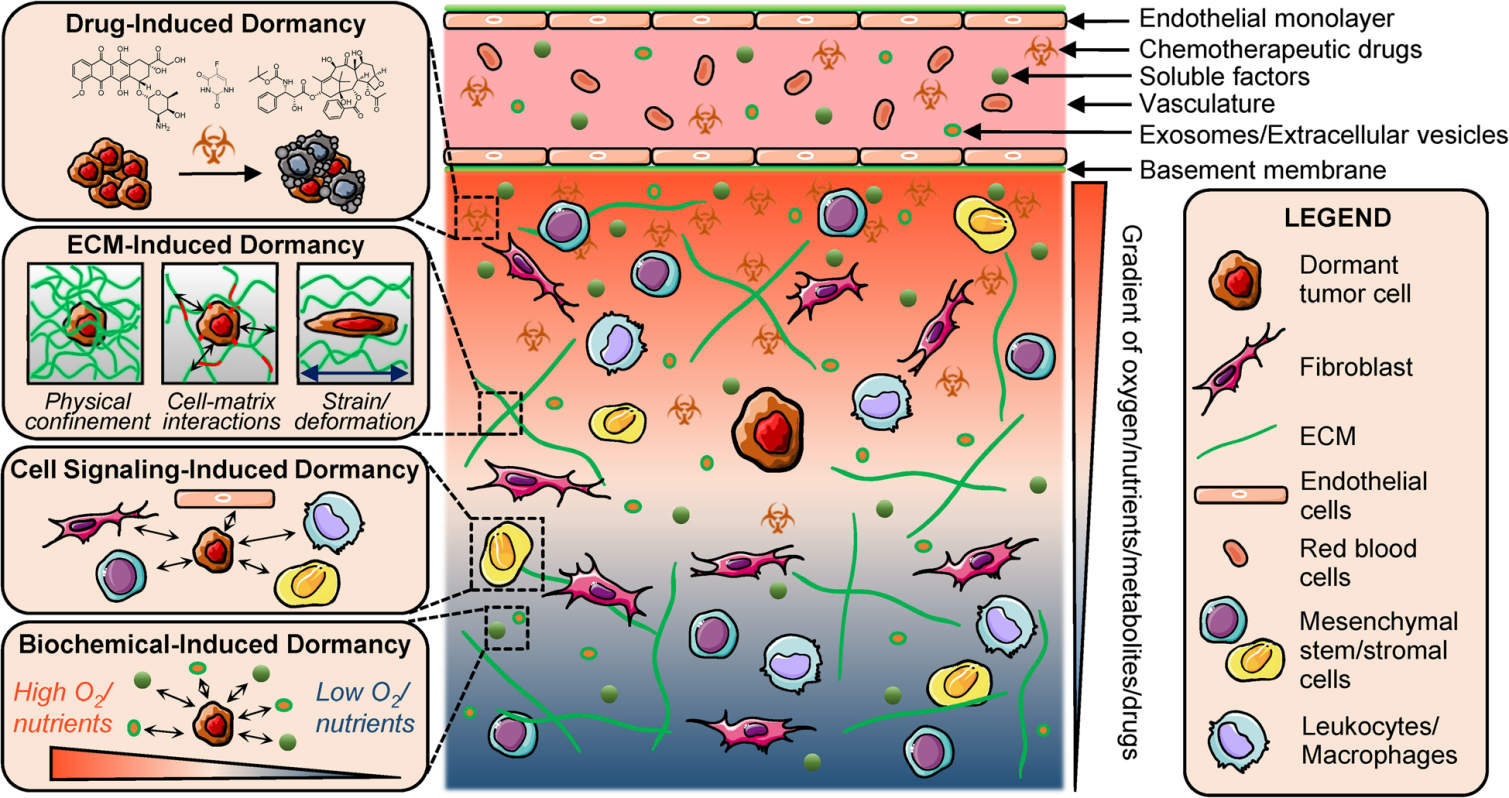
Modes of dormancy induction. Engineered, *in vitro* models of tumor dormancy can be classified based on the mode of dormancy induction: drug-induced dormancy (selective elimination and survival of sub-populations under chemotherapeutic treatment), ECM-induced dormancy (biophysical constraints imposed on cancer cells by the surrounding matrix), cell-signaling induced dormancy (paracrine signaling from stromal cells and vasculature) and biochemical-induced dormancy (influence of soluble factors, hypoxia and nutrients)

We classified existing engineered tumor dormancy models based on the mode of dormancy induction: 1) ECM-induced, 2) cell signaling-induced, 3) biochemical-induced and 4) drug-induced (Fig. 2). Efforts to create engineered models to investigate the influence of these various dormancy-inducing sources are discussed in detail below.

### ECM-induced dormancy

**Fig. 3:**
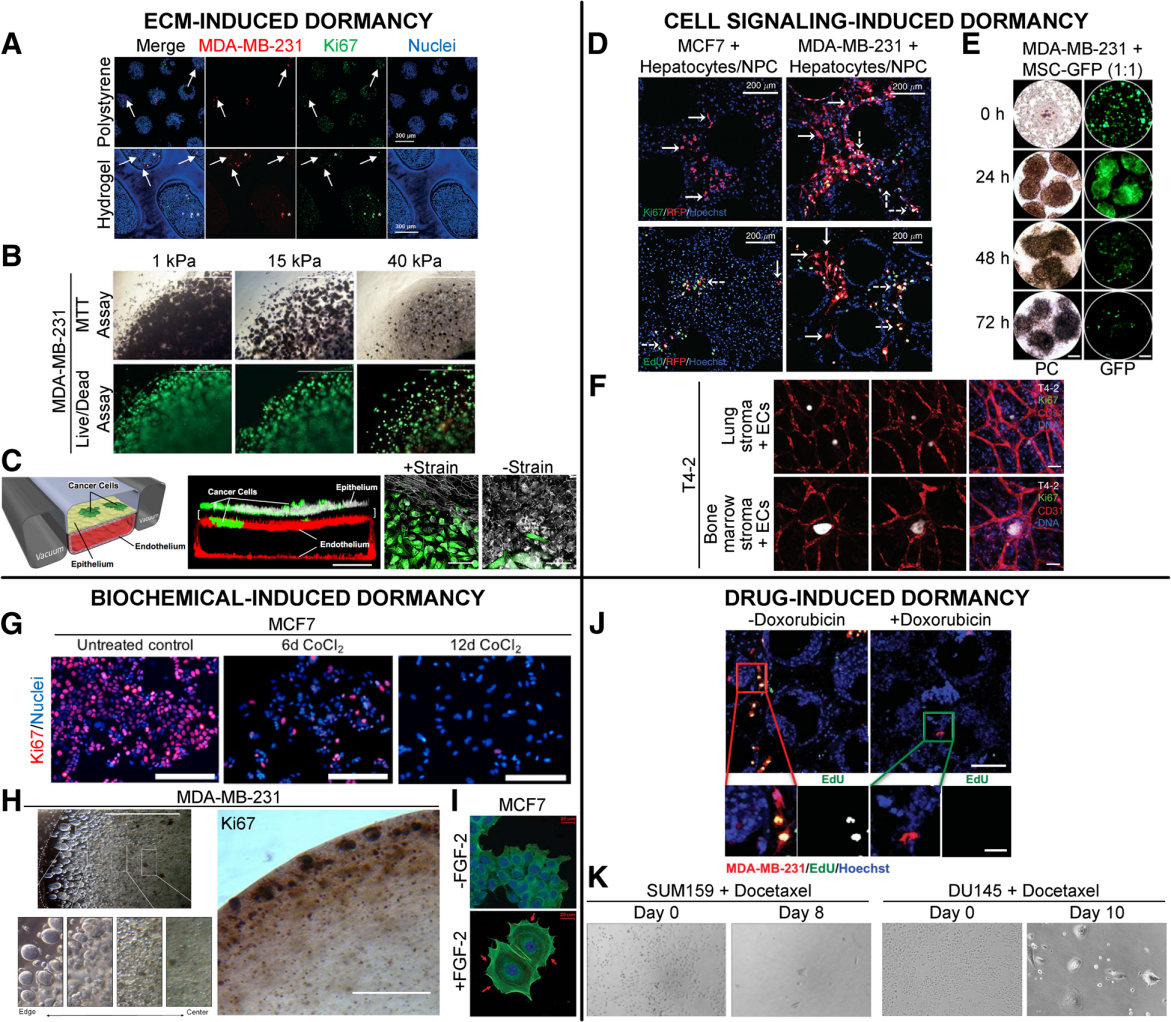
Engineered, *in vitro* models for induction of cancer dormancy. Representative examples of *in vitro* dormancy models classified by induction mode. **a** MDA-MB-231-RFP cells co-cultured with primary human hepatocytes and non-parenchymal cells (NPCs) within a hepatic microphysiological system either seeded on a polystyrene surface or encapsulated within a PEG-peptide hydrogel matrix and imaged on day 15. Arrows: dormant cells, asterisks: proliferative cells. Scale bar = 300 μm. (Adapted from [105]). Copyright 2017, RSC. **b** MDA-MB-231 cells cultured within Col-Tgel hydrogels demonstrate an increased dormancy signature characterized by reduced MTT staining, reduced cell death and lower cell density. Green: calcein AM, red: ethidium homodimer. Scale bar = 1000 μm. (Adapted from [89]). Copyright 2017, Springer Nature. **c** GFP expressing, non-small-cell lung cancer cells (NSCLC) cultured with alveolar epithelial cells and lung microvascular endothelial cells within a microfabricated lung-on-a-chip device for 2 weeks to investigate the role of physiological breathing motions on the growth/dormancy of cancer cells. Red: VE-cadherin, white: ZO-1 tight junctions, Scale bar = 200 μm (center), 50 μm (right). (Adapted from [104]). Copyright 2017, Elsevier. **d** RFP expressing breast cancer cells cultured with hepatocytes and NPCs within a liver microphysiological system for 2 weeks and fluorescently labeled for Ki67 or EdU (green) and nuclei (blue). Scale bar = 200 μm. Solid white arrows: dormant cells, dashed white arrows: proliferative cells. (Adapted from [119]). Copyright 2014, NPG. **e** MDA-MB-231 cells cultured with GFP expressing MSCs and imaged under phase contrast (PC) and green fluorescence (GFP) at varying time points are observed to cannibalize MSCs within 3D spheroids and enter dormancy, leading to reduced GFP signal intensity. Scale bar = 100 μm. (Adapted from [117]). Copyright 2016, NAS. **f** HMT-3522-T4-2 breast cancer cells cultured with lung/bone marrow stromal cells and endothelial cells remain as dormant clusters through day 17 with low proliferation. Scale bar = 100 μm. (Adapted from [42]). Copyright 2013, NPG. **g** MCF7 cells treated with 300 μM CoCl_2_ undergo hypoxia and enter dormancy with low proliferation. Scale bar = 200 μm. (Adapted from [129]). Copyright 2018, Springer Nature. **h** MDA-MB-231 cells within Col-Tgel hydrogels exhibit reduced proliferation and cluster size with increasing distance from the hydrogel edge due to a hypoxia gradient. Scale bar = 100 μm. (Adapted from [128]). Copyright 2014, PloS. **i** MCF7 cells seeded on a fibronectin-coated substrate and treated with FGF-2 undergo a dormancy phenotype with cortical actin redistribution around the perimeter of the cytoplasm (red arrows). Scale bar = 20 μm. (Adapted from [137]). Copyright 2009, Springer. **j** MDA-MB-231 cells in an engineered liver niche treated with doxorubicin exhibit reduced proliferation compared to the control group. Scale bar = 200 μm (top), 50 μm (bottom). (Adapted from [81]). Copyright 2013, ASBMB. **k** Breast and prostate cancer cells treated with docetaxel exhibit residual tumor cells with dormancy signatures. (Adapted from [148]). Copyright 2014, PloS

The most common method of ECM-mediated dormancy induction is via physical confinement of cancer cells within dense matrices that restrict proliferation, spreading, and invasion while increasing apoptosis, thereby regulating overall tumor cell quiescence and population balance [87–89]. Cancer cells, owing to their inherent robustness, are able to survive in stressful microenvironments in a dormant state and this phenomenon is exploited *in vitro* for modeling of dormant tumor microenvironments [65, 88, 90]. Mechanical and physical confinement of single tumor cells or tumor spheroids has been achieved using several biomaterials including collagen/gelatin, Matrigel, agarose, poly(ethylene glycol) (PEG)-based hydrogels, poly(ε-caprolactone) (PCL) and interpenetrating networks (IPNs) of different materials [87–96] (Fig. 3a-c). Biomaterial-based entrapment of tumor cells is dependent on modulation of crosslinking density, pore size, matrix degradability, solid stress, matrix stiffness, or a combination of these factors. These approaches can be more effective at inducing dormancy, compared to serum starvation to induce quiescence [87, 88]. A summary of biomaterial/ECM-based approaches for inducing dormancy and their associated mechanisms is provided in Table 2.

**Table 2:** ECM-based matrices to induce dormancy

Biomaterial/ECM	Characteristics	Mechanism Inducing Dormancy
Collagen/Gelatin [89]	Naturally occurring animal-derived biopolymer with collagen/gelatin backbone crosslinked with transglutaminase	Increased stiffness resulting from increased crosslinking density of gelatin precursor
Agarose [91, 93]	Plant-derived biopolymer physically crosslinked at ambient temperature	Mechanical stress arising from a confining, non-adhesive matrix
Matrigel [94]	Mouse-tumor derived matrix consisting of collagen, laminin, elastin and growth factors amongst other components	Physical confinement in a 3D matrix
Fibrin [100, 101]	Naturally occurring biopolymer in blood obtained via crosslinking of fibrinogen with thrombin	Matrix stiffness
PEG [90]	Synthetic bio-inert polymer that can be chemically and mechanically tuned	Non-degradability and physical confinement
Silica-PEG [88]	Silicate network gel formed via hydrolysis of silicon alkoxide and condensation reaction to form a porous silica network, with PEG porogen and silica nanoparticles	Physical confinement in a non-degradable matrix
Collagen-PEG IPN [87]	Double crosslinked network of collagen and PEG with varying PEG concentrations	Physical confinement in an increasingly non-degradable matrix
PEG-protein and PEG-peptide blends [92, 105]	Covalent coupling of PEG with proteins (fibrinogen) or ECM-mimetic peptides (RGDS)	Controlled cell-matrix interactions

Abbreviations: *3D* Three-dimensional, *ECM* Extracellular matrix, *IPN* Interpenetrating network, *PEG* Poly(ethylene glycol);

Collagen has been modified in several ways to modulate the behavior of encapsulated cancer cells. These include addition of transglutaminase to increase matrix crosslinking density and stiffness, varying concentration and crosslinking pH to modulate fibril diameter, fibril length, pore size, and elastic modulus, as well as formation of IPNs with PEG to increase matrix stiffness and reduce porosity [87, 89, 97] (Fig. 3b). Interestingly, cancer cells with differing inherent characteristics (epithelial vs. mesenchymal) can display widely differing behavior even under similar matrix conditions. For example, Sapudom *et al*. showed MCF7 breast cancer cells (epithelial in nature) remained as single, rounded cells within collagen matrices with a larger fibril diameter (850 nm) and associated pore size of 5.5-11 μm, while MDA-MB-231 cells (mesenchymal in nature) remained as rounded cells in matrices with a smaller fibril diameter (550 nm) and pore size of 11 μm [97]. These differences highlight the importance of both matrix microarchitecture and the cell type being investigated to attain the desired dormant tumor cell morphology.

Regulation of cell-mediated matrix degradability via modulation of PEG composition and content has also been applied to induce dormancy [88, 90, 92, 98]. These microarchitectural changes may also induce changes in diffusion of nutrients, oxygen, and cellular metabolites which could cause changes in cancer cell behavior through secondary and potentially uncontrolled mechanisms [87, 90]. In many engineered matrices, microarchitectural characteristics are coupled with each other, making it challenging to elucidate the role of individual factors toward regulating cancer cell behavior. However, some matrices allow independent control of these parameters which led to the conclusion that solid stress imposed by the matrix, and physical restriction of tumor cells in confined matrices, is a prime driver in maintaining quiescence and dormancy [88, 91, 99]. PEG-based matrices also facilitate investigation of single cell dormancy, provided that the chosen cancer cell lines are robust enough to survive within non-degradable and non-bioactive matrices, albeit for a few days in culture [88, 90].

The principal mechanisms underlying confinement-induced dormancy are attributed to decreased proliferation, increased cell death via apoptosis and limited integrin engagement, thereby making these matrices suitable for studying balanced or tumor mass dormancy. In two related studies by Liu *et al*., stiffer fibrin gels (1000 Pa) and softer collagen gels (100 Pa) restricted tumor growth while softer fibrin gels (100 Pa) promoted tumorigenicity [100, 101]. Mechanistic investigation revealed that a stiffer environment led to nuclear translocation of Cdc42, a cytosolic mechanotransducer, promoting transcription of Tet2, epigenetic upregulation of p21 and p27 with simultaneous downregulation of β_3_ integrin. Therefore, dual regulation of cell cycle progression and cell-matrix engagement can be attributed to matrix-induced dormancy [101].

The mechanisms underlying cell death due to physical confinement have been investigated in detail and may provide clues in choosing or designing matrices to study population dormancy. In non-permissive matrices, cells within dense spheroids or near central regions of the hydrogel may be limited in nutrients and oxygen leading to hypoxia and eventual necrosis [89, 90]. In other cases, apoptosis is the common mode of cell death, which can be induced by a number of factors including restricting β_1_-integrin engagement and preventing cell spreading [91, 93, 99]. Mechanical confinement has also been observed to interfere with nuclear division geometry and orientation leading to increased mitotic delay (specifically prometaphase), asymmetric multi-polar cell division, chromosome misalignment, daughter cell aneuploidy and eventual apoptosis [102, 103].

In addition to biomaterial design, advances in microfabrication and on-chip technologies have facilitated the study of liver cancer and lung cancer dormancy which incorporate multiple microphysiological cues including regulation of integrin-engagement, fluid pressure, mechanical aeration and cyclic deformation. These approaches have provided significant insights into potential targets and drug responsiveness [104, 105] (Fig. 3c). Inherent ECM cues present in tropic niches can also induce dormancy in different cancer cell types. The review by Ghajar provides a brief synopsis of some of these cues located in the lung, bone marrow and brain perivascular niches which confer dormancy signatures on cancer cells [106]. Chief among them are osteopontin and laminin, which regulate pro-survival mechanisms and therapeutic resistance in acute lymphoblastic leukemia cells, lung cancer and glioblastoma [107–110]. Overall, intelligent design of biomaterial platforms can facilitate the investigation of factors inducing cellular quiescence and tumor dormancy with a high degree of physiological complexity and direct control over desired matrix properties.

### Cell signaling-induced dormancy

Over the past few years, there has been significant interest in recapitulating the dormant secondary milieu, particularly the bone marrow, by co-culturing bone marrow stromal cells with cancer cells. The primary reason for this approach is that the complex bone marrow microenvironment is believed to contain microniches that induce tumor dormancy for extended periods of time [35, 42, 106, 111–114]. These niches confer dormancy on cancer cells via intercellular signaling leading to growth arrest, activation of pro-survival mechanisms and anti-apoptotic mechanisms, and enhanced chemotherapeutic resistance [106, 114, 115]. In a landmark study by Ghajar *et al*., breast cancer cells were co-cultured in lung-mimetic or bone-marrow mimetic perivascular niches and thrombospondin-1 (TSP1) secreted by stable endothelial networks was observed to maintain tumor cells in a dormant, non-proliferative state, with a possible supportive role of bone morphogenetic protein 4 (BMP4) [42, 116] (Fig. 3f).

The role of various secondary cell types in the bone marrow microenvironment has been modeled in several *in vitro* studies [42, 58, 104, 117]. However, the context in which these cells (mesenchymal stem/stromal cells (MSCs), endothelial cells (ECs), and cancer cells) are co-cultured often varies, making it difficult to compare and attribute specific contributions of each cell type toward dormancy induction. For example, bone marrow MSCs co-cultured with metastatic MDA-MB-231 breast cancer cells led to the cannibalism of the MSCs by the cancer cells within 72 hours. Bartosh *et al*. demonstrated that internalization of MSCs induced the cancer cells to enter a dormant phase characterized by reduced proliferation, enhanced survival capability and increased stem cell and epithelial-mesenchymal transition (EMT) marker expression [117] (Fig. 3e). This cannibalistic behavior was also observed with A549 lung cancer cells, PANC-1 pancreatic cancer cells and PC-3 prostate cancer cells. Similarly, co-culture of PC3 cells with MSCs in either bone marrow media or endothelial media helped maintain cancer cells in a growth-arrested state but the inclusion of ECs with MSCs significantly enhanced tumor cell growth [118]. In contrast, co-culture of primary bone marrow stromal cells with breast cancer cells resulted in a supportive niche that enabled higher tumor cell proliferation and *in vivo* tumorigenesis while coculture of HS-5 bone marrow stromal cells, hFOB osteoblasts and HUVECs with cancer cells resulted in an inhibitory niche that suppressed tumor cell growth and produced avascular, dormant tumors in mice [58]. Tumor dormancy was specifically induced by HS-5 and hFOB cells but not by HUVECs. The contrasting role of ECs in regulating tumor dormancy was best demonstrated in the study by Ghajar *et al*. where stable microvascular networks helped maintained tumor dormancy but sprouting neovasculature and endothelial tip cells promoted metastatic growth via secretion of periostin (POSTN) and transforming growth factor-β (TGFβ-1). Adding to this complication, TGFβ-1 is also known to show dual properties and can both induce and inhibit tumor dormancy in a context-dependent manner [42].

In addition to the bone marrow niche, the dormant liver microenvironment has also been modeled by inclusion of hepatocytes and non-parenchymal liver cells (NPCs) (Kupffer cells, sinusoidal endothelial cells and stellate cells) with breast cancer cells within an *ex vivo* microphysiological system [81, 105, 119]. Spontaneous dormancy of MDA-MB-231 and MCF7 cells was observed when cancer cells were introduced at very low densities (ratio of hepatocytes and NPCs to cancer cells >1000:1) [119] (Fig. 3d). Interestingly, inclusion of NPCs suppressed MDA-MB-231 growth but enhanced MCF7 growth, highlighting the differences in inherent tumor cell-intrinsic characteristics. Analysis of the cell secretome revealed specific cell-type differences (MDA-MB-231 cells: increased cancer attenuator follistatin and reduced pro-inflammatory cytokines IGFBP-1, MCP-1, MIP-1α, IL-6; MCF7: increased cancer signals osteopontin, sHER-2, VEGF-A, uPA, EGF amongst others). These analyses approaches could provide both prognostic and diagnostic markers of the dormant tumor behavior that could help guide future drug discovery initiatives.

Other organotropic niches modeled for tumor dormancy studies include breast cancer, bladder cancer, prostate cancer and lung cancer [104, 120]. In some cases, tumor dormancy can be induced simply by co-culturing breast, bladder or prostate cancer with respective stromal cells/fibroblasts on an adhesion-limited substrate and coaxing the cells to form 3D spheroids as demonstrated by Pavan *et al*. [120]. However, more intricate approaches involve tri-culture and differentiation of cell types within microfabricated devices to mimic more complex physiological structures. In one model of lung cancer dormancy, human lung microvascular cells were cultured under flow to form a uniform patent lumen with a layer of differentiated human primary airway epithelial cells or human primary alveolar epithelial cells to mimic the airway epithelium or alveolar epithelium respectively. H1975 non-small cell lung carcinoma (NSCLC) cells were seeded at low densities (>100:1 epithelial cells: cancer cells) to maintain low proliferation, spreading and invasion over 3-4 weeks in culture [104]. Overall, these examples demonstrate that recapitulation of organ-specific niches that suppress tumor growth and promote tumor cell quiescence is quite achievable in engineered constructs and microphysiological systems, provided that secondary cell types are presented in the right context and environmental conditions.

In conjunction with direct cell-cell contact and cell-secreted soluble factor signaling, tumor dormancy is also mediated by exosomal- and miRNA-based regulation, specifically between bone marrow MSCs and breast cancer cells [121–126]. MSC-derived exosomes were found to be responsible for horizontal transfer of miR-23b in bone marrow-metastatic BM2 breast cancer cells, which led to suppressed proliferation, reduced stem cell marker expression, reduced matrix invasion and sensitivity to docetaxel, by suppression of the target gene *MARCKS* [122]. The effect of exosomes from naïve and tumor-educated MSCs on breast cancer cell lines has also been examined. Tumor-primed MSC exosomes were more effective at inducing cycling quiescence and G_0_/G_1_ arrest in MDA-MB-231 cells, but not T47D cells, via transfer of miR-222/223 [121]. Exosomes derived from poorly-metastatic tumors can also initiate an innate immune response via recruitment of patrolling monocytes, NK cells and macrophages leading to suppression of distant metastasis [127].

These differences highlight the complex nature of inter-cellular interactions through parallel mechanisms and inherent tumor-intrinsic variations, which makes it challenging to generalize or predict molecular mechanisms underlying tumor dormancy. Engineered *in vitro* systems may provide the opportunity to elucidate the underlying mechanisms that mediate dormancy induction via direct cell-cell contact or soluble factors secreted from secondary cells.

### Biochemical-induced dormancy

Modulation of the secondary organ-mimetic milieu via biochemical cues has been applied toward induction of tumor dormancy in several studies. These cues include induction of hypoxia, inhibition of nutrient diffusion, and addition/removal of soluble factors, either to inhibit cell-intrinsic pro-tumorigenic mechanisms or to inhibit downstream cell-cell and cell-matrix interactions [92, 128, 129]. Hypoxia plays a ‘Janus’-like role in the tumor dormancy program, particularly in angiogenic dormancy. Chronic or diffusion-limited hypoxia can lead to increased expression of vascular endothelial growth factor (VEGF), TGF-β, platelet derived growth factor (PDGF), urokinase-type plasminogen activator (uPA) and other factors which upregulate angiogenic sprouting, promote matrix invasion and ultimately lead to metastatic growth. However, hypoxia can also induce apoptosis via multiple hypoxia inducible factor-1α (HIF-1α) dependent and independent pathways, which may regulate single cell dormancy [130, 131]. Hence, finding the optimum balance between these opposing processes in a context-dependent manner is necessary to maintain tumor dormancy in *in vitro* models.

Interestingly, some cancer cells may enter into a hypoxia-mediated dormancy program to survive in a stressful microenvironment. AsPC-1, a pancreatic cancer cell line, when maintained under chronic 1% O_2_ hypoxia underwent reduced proliferation, cell death and ATP turnover with an altered AKT-dependent metabolic program while MDA-MB-231 cells underwent G_0_/G_1_ arrest with reduced metabolism and increased expression of stem-cell markers [132, 133]. Hypoxia in the primary tumor microenvironments of human head and neck squamous cell carcinoma (HNSCC) and triple negative breast cancers (TNBC) can also give rise to a subset of dormant cells in mouse, patient-derived xenografts (PDXs) and human tumors [134]. Induction of hypoxia can be achieved in a number of different ways: addition of iron-binding/substituting agents that inhibit HIF-1α degradation and stabilize its cytosolic expression, and imposition of diffusion-limited hypoxic gradients (Fig. 3h). In one study, microfabricated nano-intravital devices (iNANIVIDs) loaded with desferrioxamine were implanted in T-HEp3 tumors grown in a CAM model to induce hypoxia from 4 hours to 3 days post-implantation [134]. Alternatively, cobalt chloride added to culture media of MCF7, MDA-MB-231 and OVCAR-3 cells induced hypoxia and resulted in similar dormancy responses as cells maintained in 0.1% O_2_ conditions in both 2D culture and 3D collagen gels [129] (Fig. 3g). Breast, prostate and colon cancer cells encapsulated in Col-Tgel (collagen crosslinked with transglutaminase) hydrogels underwent diffusion-limited hypoxia over 9 days imposed by the 3D matrix which led to restricted cell proliferation, smaller clusters and cellular quiescence/necrosis [128]. Overall, hypoxia-mediated induction of the tumor dormancy program is a potential approach for investigating dynamic regulation occurring in both primary and disseminated tumor microenvironments.

Aside from hypoxia, soluble factor-mediated regulation of tumor dormancy has also been investigated. The most common approach is limiting growth factor mediated signaling in tumor cells by culturing cells in serum-free or low-serum media [87, 88]. Although this method does induce cellular quiescence and restricted growth, the exact factors mediating this response can be difficult to elucidate. Fibroblast growth factor-2 (FGF-2), found abundantly expressed in the bone marrow stroma, has been used to induce dormancy in several breast cancer cell lines [135–140] (Fig. 3i). Barrios *et al*. found that FGF-2 regulates partial re-differentiation in some breast cancer cell lines, reduces motility and invasion, upregulates α_5_β_1_ integrin expression and induces pro-survival characteristics through the PI3K/AKT signaling pathway. α_5_β_1_ integrin ligation with fibronectin in the bone marrow stroma also independently regulated tumor dormancy [137]. Other factors found to induce tumor dormancy include 5-azadeoxycytidine (5-Aza-C), a DNA methylating agent, and retinoic acid, as shown by Sosa *et al*. [141]. The vitamin A-retinoic acid complex is known to regulate dormancy of hematopoietic stem cells in the bone marrow niche [142, 143]. An ‘epigenetic therapy’ of 5-Aza-C and retinoic acid was found to upregulate a master receptor, NR2F1, which induced quiescence in cancer cells via upregulation of pluripotency genes *SOX9*, *RARβ* and *NANOG* [141]. Administration of specific anti-angiogenic agents (e.g. angiostatin, thrombospondin) could also limit angiogenic growth near dormant tumor sites and prolong pre-angiogenic dormancy [42, 144, 145]. These strategies could potentially be adopted in *in vitro* dormancy models, keeping in mind the context-dependency of tumor cell lines.

### Drug-induced dormancy

Therapy-induced tumor dormancy has been a rising challenge in addressing metastatic recurrence due to the chemoresistant nature of dormant tumor cells [73, 146]. Drug treatment of cancer cells *in vitro* and *in vivo* has been conducted in several studies to model therapy-induced dormancy observed in clinical settings [81, 95, 134, 147, 148]. Chemotherapeutics known to induce tumor dormancy *in vivo* and in patients include doxorubicin, docetaxel, cyclophosphamide, 5-fluorouracil (5-FU), methotrexate, cisplatin, bevacizumab, and trastuzumab [12, 149–153]. Similarly, some of these drugs used in *in vitro* models include doxorubicin, docetaxel, 5-FU, carboplatin amongst others [81, 95, 147, 148, 150] (Fig. 3j-k). The effect of doxorubicin administered to MDA-MB-231 cells in an engineered hepatic niche was evaluated by comparative analysis of the cytokine profile of growing, and dormant tumor populations, which revealed important similarities (Fraktalkine, RANTES, MCP-2, GM-CSF) and differences (VEGF-A, IL-12p70, IL-7, PECAM-1) in expression levels [81]. Associated effects of drug-induced tumor dormancy include enrichment of cells expressing stem-cell like characteristics and other clonal sub-populations, enhanced survival upon removal of treatment and an imbalance of pro- and anti-survival mechanisms [147, 148, 152].

Interestingly, metronomic chemotherapy regimens (drugs administered at regular, frequent doses to maintain a low, but active, range of drug concentrations over long time periods at low toxicity levels) have been proposed as an approach to induce angiogenic tumor dormancy [153–156]. Drugs that suppress HIF-1α expression (e.g. topotecan, irinotecan, Adriamycin) or VEGF expression (irinotecan, 5-FU, oxaliplatin, paclitaxel and docetaxel) in different cancer cell lines could possibly be administered metronomically to suppress angiogenic outgrowth and prolong angiogenic dormancy. Overall, several strategies for inducing dormancy programs via drug treatment remain to be explored and adopted in *in vitro* engineered models.

## *In vitro* models of metastatic recurrence/relapse

**Fig. 4:**
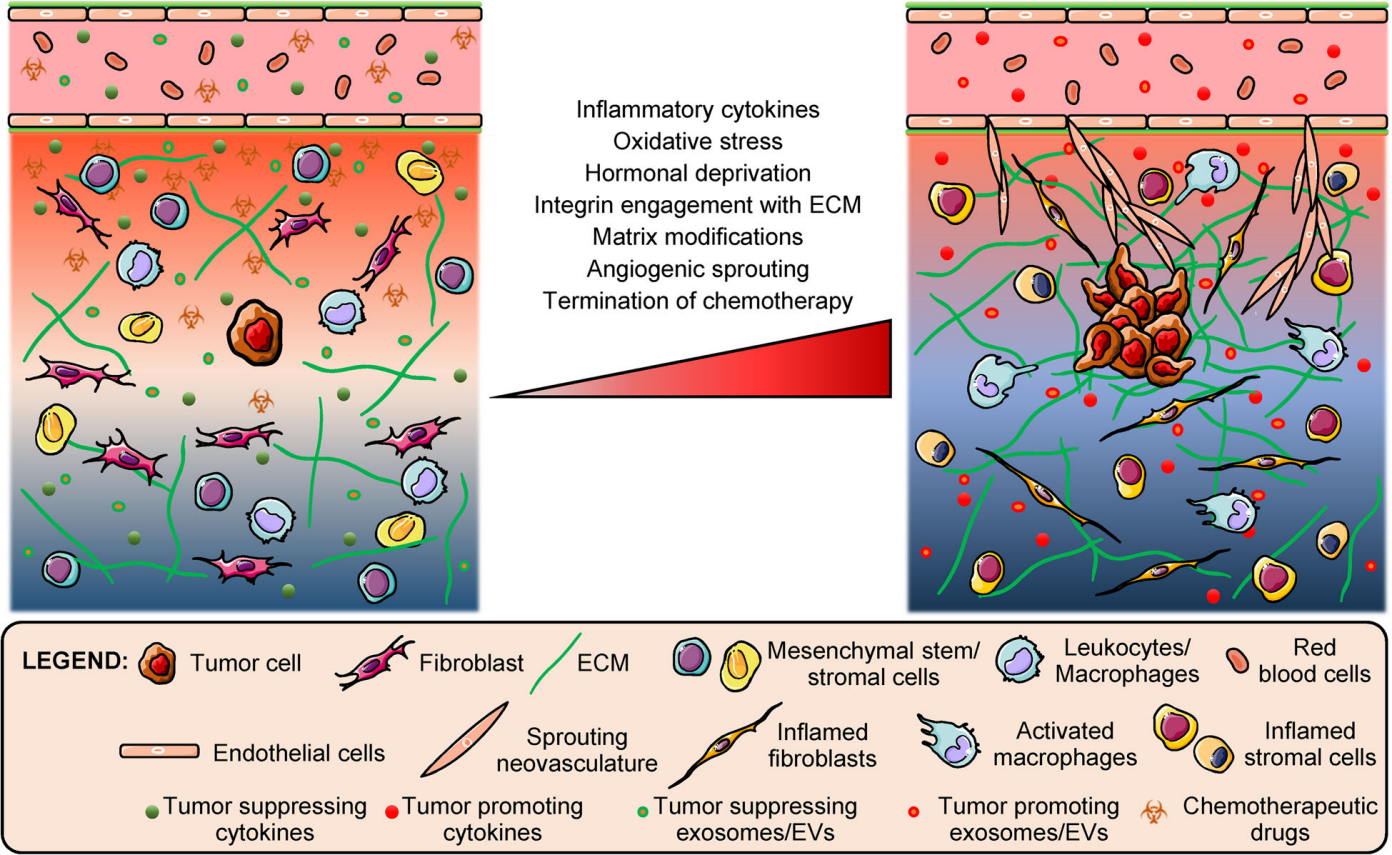
Factors influencing reactivation of dormant cancer cells. Dormant tumor cells in the secondary niche can be stimulated or triggered toward metastatic growth via multiple sources including pro-inflammatory and angiogenic factors, paracrine signaling by stromal cells and sprouting vasculature, and dysregulated cell-matrix interactions amongst others

The main challenge in preventing metastatic recurrence or relapse is the presence of drug-tolerant persister sub-populations and quiescent cells that exhibit a high degree of EMT plasticity and cancer stemness, thereby enabling survival under stressful and harsh microenvironments through activation of pro-survival mechanisms [157–159]. Very often, clonal populations expanded from these drug-selected sub-populations exhibit a higher degree of chemoresistance and need to be targeted via alternate mechanisms. Multiple factors including stromal and endothelial cell signaling and surgery-induced inflammation have been implicated toward triggering the metastatic growth from dormant tumor cells [9, 160, 161] (Fig. 4). Several *in vitro* models simulating this phase of tumor evolution have been developed and various techniques have been employed to induce reactivation of 3D encapsulated dormant cancer cells.

One prominent approach of studying metastatic relapse using *in vitro* biomaterial-based models is through spatiotemporal modulation of the engineered matrix [88, 89, 162, 163]. Modulation of matrix characteristics can be achieved by partial enzymatic digestion of a confining restrictive matrix, thereby lowering stiffness and crosslinking density and allowing encapsulated dormant cells more freedom to proliferate and invade the surrounding matrix [89]. Alternatively, cells can be completely extracted from the confining matrix (and potentially re-embedded in a soft, permissive matrix) to induce a proliferative switch from a quiescent state [88, 89]. 3D spheroids containing dormant tumor cells, when transferred to a more adhesive substrate, demonstrated higher cell dissemination and spreading [120]. Integrin engagement of dormant cancer cells with specific ECM proteins (fibronectin, versican, tenascin-C, collagen-I) can also promote metastatic relapse [42, 162]. Barkan *et al*. demonstrated that dormant D2.0R mouse mammary cancer cells overexpressing integrin β_1_ in a collagen-I-rich fibrotic matrix leads to phosphorylation of SRC, FAK and MLC, activation of ERK, actin stress fiber formation, and cancer cell spreading [162]. These studies demonstrate that multiple approaches can be employed to modulate matrix characteristics ultimately leading to activation of proliferation in dormant cancer cells.

Other approaches for investigating the dormancy-proliferation switch involve direct stimulation of dormant tumor cells via pro-inflammatory cytokines and angiogenic growth factors, indirect paracrine signaling from activated/inflamed secondary cells, and termination of chemotherapeutic/hypoxic challenge amongst others [42, 58, 59, 81, 129, 139, 140, 148, 164]. Some common pro-inflammatory cytokines used to induce proliferation and invasion of cancer cells include lipopolysaccharide (LPS), epidermal growth factor (EGF), tumor necrosis factor α (TNFα), interleukin β (IL-β), interleukin 6 (IL-6) and prostaglandin E2 (PGE2) [81, 139]. Insulin-like growth factor 1 (IGF1) was found to initiate self-renewal of lung cancer stem cells in dormant lung tumors via the activation of a PI3K/Akt/β-catenin pathway and production of the angiogenic factors chemokine (C-X-C motif) ligand 1 (CXCL1) and placental growth factor (PlGF) [59]. Periostin and TGFβ-1 secretion from sprouting neovasculature promoted metastatic growth in dormant colonies of breast cancer cells in engineered lung and bone marrow stromal matrices [42]. Small molecule inhibition of specific factors (p38 MAPK, Alk5 and receptor tyrosine kinases) associated with dormancy pathways have also been used to reverse the inhibition of cell-cycle arrest in quiescent cancer cells and promote proliferation [58].

Stromal cells, activated by pro-inflammatory cytokines, oxidative stress or estrogen deprivation, can indirectly stimulate dormant cancer toward a proliferative state [139]. Paracrine signaling from stromal cells can also occur via horizontal transfer of mitochondrial DNA (from cancer-associated fibroblasts) via extracellular vesicles (EVs), induction of oxidative phosphorylation and an exit from dormancy [164]. Considering that exosomes and EVs from the primary tumor and secondary niche cells can regulate the pre-metastatic niche, favoring tumor growth, they can also be surmised to influence the dormancy-proliferative switch via undiscovered mechanisms [165–170]. Multiple approaches for investigating the dormancy/proliferation switch exist and they can be applied in a context dependent-manner in engineered *in vitro* models to investigate the molecular mechanisms underlying tumor recurrence and provide potential targets for therapeutic intervention.

## Therapeutic strategies for tumor dormancy

A majority of FDA approved anti-cancer therapeutics are targeted towards inhibiting cell proliferation, inducing cell cycle arrest and cell death [171]. However, dormant tumor cells (exhibiting low proliferation, cellular quiescence, high clonal heterogeneity) can be difficult to treat using these therapeutics. One comparative study demonstrated that conventional drugs including paclitaxel, doxorubicin, and 5-FU eliminate 2D cultured cells with high Ki67 expression; but lose their respective efficacy against the same cell types when cultured as 3D spheroids [172]. Additionally, diverse drug-resistance mechanisms can evolve from individual persister drug-tolerant cells, thereby necessitating synergistic targeting approaches for effective treatment of heterogeneous clones [157]. Hence, efforts are underway to discover novel targets, signaling pathways and therapeutic strategies to treat slow-cycling sub-populations and minimal residual disease as pre-emptive measures to eliminate dormant tumor cells [86, 173–175].

Toward this end, high-throughput drug screening studies have revealed potential mechanisms employed by cancer cells to survive chemotherapeutic insult and to develop alternate targeting strategies to enhance quiescent cell death [176–178]. MDA-MB-231 cells (in co-culture with HS-5 bone marrow stromal cells) treated with doxorubicin were able to survive through compensatory action of the MEK pathway and Cavnar *et al*. demonstrated that use of MEK inhibitors as synergistic agents selectively induced death in cancer cells compared to stromal cells [179]. A drug response-based gene expression profiling study on colon cancer cell lines revealed that quiescent cells in 3D spheroids exhibit upregulated cholesterol biosynthesis and mevalonate pathway genes that can be synergistically targeted with statins (simvastatin, Atorvastatin: cholesterol-lowering drugs, inhibitors of the mevalonate pathway) and oxidative phosphorylation inhibitors (nitazoxanide, salinomycin, antimycin A, FCCP, oligomycin A) [178]. Similarly, using respiratory chain inhibitors (metformin, antimycin A) against breast, prostate, and colon cancer spheroids in conjunction with cytostatic agents (paclitaxel, cisplatin) helped eliminate proliferative as well as dormant sub-populations within the spheroids, leading to low spheroid viability [177]. Interestingly, co-administration of cytochalasin B or 2-deoxy-D-glucose, inhibitors of cellular glucose uptake or glycolysis respectively, led to complete death of tumor spheroids indicating that glucose concentration in the surrounding microenvironment also confers some degree of resistance [177]. Another high throughput screening study revealed two potential hits against dormant micrometastasis in MDA-MB-435 cells grown on SISgel (obtained from ECM of small intestine submucosa) [176]. An extensive algorithmic search of similar compounds revealed potential targets which include matrix metalloproteinases, protein-tyrosine phosphatase, carbonic anhydrases and adenosine A1/A2/A3 receptors amongst others. Inducing chronic endoplasmic reticulum (ER) stress using thapsigargin (a SERCA inhibitor) combined with bortezomib (a proteasome inhibitor) also caused significant cell death in dormant breast and bladder cancer spheroids via protein misfolding and inhibition of an anti-apoptotic survival pathway [120]. Subsequent RNA-sequencing of dormant cells revealed upregulated ribosomal protein genes (protein translation) and pro-apoptotic protein-coding genes which could also provide additional targets for future dormancy-specific drugs [120].

Although high-throughput drug screening has traditionally been conducted on 2D cultured cells or 3D spheroids in well plates, the translation of novel *in vitro* dormancy models to a high-throughput format is of great interest. The high degree of control and uniformity presented in simplistic *in vitro* models make them amenable for high-throughput analysis. However, incorporation of complex elements of the dormant niche (including stromal cells, endothelial cells, ECM proteins, growth factors) could potentially prove challenging with respect to scale-up. Toward this goal, Kenny *et al*. demonstrated screening of >2400 drug compounds against metastatic ovarian cancer cells in a 3D organotypic culture that included mesothelial cells and fibroblasts in a fibronectin and collagen I-rich ECM [180]. A similar approach could potentially be extended for use with other engineered biomaterials and existing platforms. However, integration of microfluidic organ-on-a-chip systems with established high-throughput screening platforms is still challenging owing to technical complexities of maintaining dynamic fluidic perfusion, continuous and end point readouts, and high variability in dynamic culture systems.

Targeting cell-matrix interactions in dormant niches has also been tested with one study reporting that administration of flavopiridol selectively abrogated dormant clones of MCF7 and T47D breast cancer cells via suppression of integrins α_5_ and β_1_, reduced adhesion to fibronectin, diminished Akt phosphorylation and total protein levels of ERK1/2 and p38 [181, 182]. Additional treatment with MEK inhibitors or p38 inhibitors caused further reduction in dormant clones for both cell types, indicating that multiple pathways need to be targeted in parallel to achieve reasonable efficacies [181]. Inhibition of ERK1/2 phosphorylation, MAPK signaling, suppression of uPA receptor expression along with upregulation of p38α/β expression and phosphorylation could be adopted as a strategy for inducing dormancy programs in multiple cancer cell types [183–185].

Additional therapeutic approaches toward preventing reactivation of dormant cells, metastatic relapse, and prolonging of the dormancy state are also being developed. Most prominent amongst them are suppression of cell-matrix interactions promoting adhesion, invasion and migration, EMT, angiogenic growth, inflammatory signaling, cancer stemness, and immunoediting of specific immune cell types (NK cells, myeloid derived suppressor cells) [72, 186–189]. For example, an *in silico* phenotype screen against several breast cancer cell lines identified alprostadil and haloperidol as anti-metastatic agents capable of reducing membrane fluidity, cell motility and resulting EMT [186]. Some candidate agents which could potentially be used to prolong dormancy programs include metarrestin [190], canakinumab [188], cabozantinib [189], and metformin [191, 192], along with other repurposed FDA-approved drugs and those in clinical trials for tumor recurrence (as reviewed by Hurst *et al*.) [86]. The reviews by Ordóñez-Morán and Dittmer summarize key promising dormancy-associated targets in the complex metastatic microenvironment niches that have been verified in cancer cell lines and animal models with potential translatability to humans [72, 182]. Some of these targets include the SRC family of kinases, STAT3, β_1_ integrin, VCAM-1, CXCR4, JAG1, TGFβ3, and periostin amongst others. Modulation of metastasis suppressor proteins (chief among them BRMS1, KISS1) and associated genes could also provide a therapeutic strategy against metastatic relapse in multiple cancer types [53, 72, 75, 193].

From pre-clinical observations, targeting of pre-metastatic disease and dormant tumor cells appears promising and offers a longer window of opportunity than intervention therapy for overt disease. Further, targeting dormant clones may prevent these cells from establishing a micrometastatic niche and isolated dormant cells could be more vulnerable at this stage. However, from a clinical perspective, operating such proposed metastasis prevention and anti-dormancy trials in the adjuvant setting may be difficult owing to several challenges. Such trials would involve long-term monitoring of large cohorts of patients, which may exceed the regulatory patent protection periods. Enrollment of patients in cohorts needs to be carefully evaluated to identify those that are most at risk of recurrent disease. Current endpoints for cancer treatment need to be reconsidered to incorporate long-term patient benefits, safety and efficacy specifically against dormant cells and time to metastasis, rather than tumor shrinkage [182]. The review by Goddard *et al*. provides a summary of clinical trials pertinent to tumor dormancy including targeting agents for DTCs and dormancy-specific end point metrics [194].

Overall, substantial opportunities exist for discovery of dormancy-associated targets and employing *in vitro* models may significantly enhance the capability of screening large numbers of potential compounds. These models may also provide mechanistic insight into dormancy mechanisms that could be exploited to test the efficacy of different compounds against quiescent, slow-cycling cells and thereby strengthen the repertoire of the drug discovery pipeline.

## Conclusions and future perspectives

Overall, the significance of tumor dormancy and metastatic relapse in the context of cancer research and treatment has been discussed. The lack of, and the need to develop, engineered, *in vitro* models of tumor dormancy has been presented. Current approaches adopted for modeling of tumor dormancy and metastatic relapse using engineered biomaterials and microfabrication techniques have been described. Some of the key mechanisms associated with dormancy and potential targets discovered using these *in vitro* models have also been presented.

Biomaterial-based cancer models have mostly been limited to investigation of tumorigenesis and metastasis thus far. However, the importance of tumor dormancy and metastatic recurrence is gradually becoming more apparent among the tissue engineering and biomaterials communities. Accordingly, more focus is being directed toward modeling of tumor dormancy via recapitulation of cellular quiescence, cell cycle arrest, and low proliferation within engineered platforms. However, significant gaps in knowledge still exist with regards to the molecular mechanisms governing tumor dormancy, variation among cancer types, contextual dependency with stromal microenvironments, and definition of standard biomarkers or cell states associated with the dormant phenotype. One major aspect of tumor dormancy that has been underexplored *in vitro* is the role of immune cells in regulating dormancy mechanisms and imbalances in immune regulation that may help tumor cells escape dormancy. With recent advances in engineering pre-metastatic niches and immune cell engineering, these cell types could be incorporated in biomaterial-based models to recapitulate immune-regulated tumor dormancy [110]. Humanized, implantable engineered niches that can be serially transplanted in mice and used for long-term investigation of disseminated tumor cells along with interactions with immune and stromal cells can provide significant insight into mechanisms of dormancy as well as reactivation [195].

As further investigations and discoveries in tumor dormancy biology are made over time, it is expected that *in vitro* engineered models will also be improved concurrently to recapitulate these disease states with a high degree of physiological context. These models could provide additional tools and platforms to biologists that could potentially culminate in development of dormancy-targeted drugs, thereby improving survival outcomes in patients.

## Abbreviations

2D: Two-dimensional
3D: Three-dimensional
5-Aza-C: 5-Azadeoxycytidine
5-FU: 5-Fluorouracil
AKT: Protein Kinase B
ATP: Adenosine Triphosphate
BMP4: Bone Morphogenetic Protein 4
BRMS1: Breast cancer-Metastasis Suppressor 1
CAM: Chick Chorioallantoic Membrane
Cdc42: Cell Division Control Protein 42 homolog
CTC: Circulating Tumor Cell
CXCL1: Chemokine (C-X-C Motif) Ligand 1
CXCR4: C-X-C Chemokine Receptor 4
EC: Endothelial Cell
ECM: Extracellular Matrix
EdU: 5-Ethynyl-2’-Deoxyuridine
EGF: Epidermal Growth Factor
EMT: Epithelial-Mesenchymal Transition
ER: Endoplasmic Reticulum
ERK: Extracellular Regulated Kinase
EV: Extracellular Vesicles
FAK: Focal Adhesion Kinase
FCCP: Carbonyl Cyanide 4-(Trifluoromethoxy) Phenylhydrazone
FDA: Food and Drug Administration
FGF-2: Fibroblast Growth Factor 2
GFP: Green Fluorescent Protein
GM-CS: Granulocyte-Macrophage Colony-Stimulating Factor
HIF-1α: Hypoxia Inducible Factor-1α
HNSCC: Head and Neck Squamous Cell Carcinoma
HUVEC: Human Umbilical Vein Endothelial Cell
IGF1: Insulin-like growth factor 1
IGFBP-1: Insulin Like Growth Factor Binding Protein 1
IL-6/7/12/β: Interleukin 6/7/12/β
iNANIVID: Nano-Intravital Device
IPN: Interpenetrating Network
JAG1: Jagged1
LPS: Lipopolysaccharide
MAPK: Mitogen Activated Protein Kinase
MCP-1: Monocyte Chemoattractant Protein 1
MCP-2: Monocyte Chemoattractant Protein 2
MEK: Mitogen-Activated Protein Kinase Kinase
MIP-1α: Macrophage Inflammatory Protein 1α
miRNA: MicroRNA
MLC: Myosin Light Chain
MSC: Mesenchymal Stem/Stromal Cell
MTT: 3-(4,5-Dimethylthiazol-2-yl)-2,5-Diphenyltetrazolium Bromide
NK: Natural Killer Cell
NPC: Non-Parenchymal Cell
NSCLC: Non-Small Cell Lung Carcinoma
PCL: Poly (ε-Caprolactone)
PDGF: Platelet Derived Growth Factor
PDX: Patient Derived Xenograft
PECAM-1: Platelet Endothelial Cell Adhesion Molecule 1
PEG: Poly(Ethylene Glycol)
PGE2: Prostaglandin E2
PI3K: Phosphoinositide 3-Kinase
PlGF: Placental Growth Factor
POSTN: Periostin
RANTES: Chemokine (C-C motif) Ligand 5
RFP: Red Fluorescent Protein
SERCA: Sarco/Endoplasmic Reticulum Ca^2+^-ATPase
sHER-2: Soluble Human Epidermal Growth Factor Receptor 2
SRC: Proto-Oncogene Tyrosine-Protein Kinase
STAT3: Signal Transducer and Activator of Transcription 3
Tet2: Tet Methylcytosine Dioxygenase 2
TGF-β: Transforming Growth Factor β
TNBC: Triple Negative Breast Cancer
TNFα: Tumor Necrosis Factor Α
uPA: Urokinase-Type Plasminogen Activator
VCAM-1: Vascular Cell Adhesion Molecule 1
VE-cadherin: Vascular Endothelial Cadherin
VEGF-A: Vascular Endothelial Growth Factor A
ZO-1: Zona Occludens 1
